# The Effects of Andrographolide on the Enhancement of Chondrogenesis and Osteogenesis in Human Suprapatellar Fat Pad Derived Mesenchymal Stem Cells

**DOI:** 10.3390/molecules26071831

**Published:** 2021-03-24

**Authors:** Thitianan Kulsirirat, Sittisak Honsawek, Mariko Takeda-Morishita, Nuttanan Sinchaipanid, Wanvisa Udomsinprasert, Jiraporn Leanpolchareanchai, Korbtham Sathirakul

**Affiliations:** 1 Department of Pharmacy, Faculty of Pharmacy, Mahidol University, Bangkok 10400, Thailand; lordrx16@gmail.com (T.K.); jiraporn.lea@mahidol.ac.th (J.L.); 2 Osteoarthritis and Musculoskeleton Research Unit, Department of Biochemistry, Faculty of Medicine, Chulalongkorn University, Bangkok 10330, Thailand; sittisak.h@chula.ac.th; 3 Laboratory of Drug Delivery Systems, Faculty of Pharmaceutical Sciences, Kobe Gakuin University, Kobe, Hyogo 650-8586, Japan; mmtakeda@pharm.kobegakuin.ac.jp; 4 Department of Manufacturing Pharmacy, Faculty of Pharmacy, Mahidol University, Bangkok 10400, Thailand; nuttanan.sin@mahidol.ac.th; 5 Department of Biochemistry, Faculty of Pharmacy, Mahidol University, Bangkok 10400, Thailand; wanvisa.udo@mahidol.ac.th

**Keywords:** mesenchymal stem cells, suprapatellar fat pad, andrographolide, osteoarthritis, regenerative medicine

## Abstract

Andrographolide is a labdane diterpenoid herb, which is isolated from the leaves of *Andrographis paniculata,* and widely used for its potential medical properties. However, there are no reports on the effects of andrographolide on the human suprapatellar fat pad of osteoarthritis patients. In the present study, our goal was to evaluate the innovative effects of andrographolide on viability and Tri-lineage differentiation of human mesenchymal stem cells from suprapatellar fat pad tissues. The results revealed that andrographolide had no cytotoxic effects when the concentration was less than 12.5 µM. Interestingly, andrographolide had significantly enhanced, dose dependent, osteogenesis and chondrogenesis as evidenced by a significantly intensified stain for Alizarin Red S, Toluidine Blue and Alcian Blue. Moreover, andrographolide can upregulate the expression of genes related to osteogenic and chondrogenic differentiation, including *Runx2*, *OPN*, *Sox9*, and *Aggrecan* in mesenchymal stem cells from human suprapatellar fat pad tissues. In contrast, andrographolide suppressed adipogenic differentiation as evidenced by significantly diminished Oil Red O staining and expression levels for adipogenic-specific genes for *PPAR-γ2* and *LPL*. These findings confirm that andrographolide can specifically enhance osteogenesis and chondrogenesis of mesenchymal stem cells from human suprapatellar fat pad tissues. It has potential as a therapeutic agent derived from natural sources for regenerative medicine.

## 1. Introduction

Osteoarthritis (OA) is one of the most common debilitating diseases encountered globally. The clinical symptoms of OA are pain and stiffness in joints. It affects women more than men [1]. 80% of patients with OA have some degree of movement impairment. This leads to diminished performance in the workplace, and 25% of patients cannot perform the main activities of their daily life, which often leads to social isolation and depression [2]. Despite decades of drug research and development, no disease-modifying drug for osteoarthritis has been approved for use in humans [3]. Such a drug could slow progression by reducing the rate of cartilage degeneration. Nowadays, available pharmacotherapies focus on pain relief only after symptoms are present, by the use of acetaminophen, cyclooxygenase-2 (COX2) inhibitors and corticosteroids.

One of the hallmarks of OA is the progressive loss that occur in the articular and subarticular cartilage structures. The early stage in the course of the disease is a significantly involves a degradation and re-modelling (both bone formation and resorption) of the bone cartilage interface, especially in areas underlying damaged cartilage. The trabecular bone and the cortical subchondral plate thickens and becomes increasingly irregular [4]. The biomechanical factors, such as loading during weight bearing on the subarticular bone beneath areas of damaged cartilage, and pathobiochemical influences, such as enhanced release of tissue growth factors and cytokines [5]. The primary cytokines and enzymes responsible to catabolic processes include, interleukin-1β (IL-1β) and matrix metalloproteinases (MMPs), growth factors and free radicals, among others [6]. Bone morphogenetic proteins (BMPs) and insulin growth factor 1 (IGF1) are endogenous anabolic factors that stimulate cartilage generation and remodelling [7,8]. In addition, health problems of excessive osteoclast formation and bone resorption can cause an imbalance in bone remodeling and thus induce many adult skeletal diseases, including osteoporosis, rheumatoid arthritis, osteoarthritis, multiple myeloma, metastatic cancers, premature menopause, low levels of testosterone and estrogen in men, all potentially leading to changes in cartilage homeostasis.

Herbal remedies have been used in traditional medicine practices for centuries. It has shown a promising alternative improvement of patient conditions and also could alleviate a wide range of diseases [9]. *Andrographis paniculata* (Burm.f.) Wall. ex Nees (AP) belongs to the family Acanthaceae. This plant is known as “king of bitters” for its extremely bitter taste. It is a well-known traditional medicinal plant in Ayurveda of India, Bangladesh, Thailand, and China [10,11]. Whole plant leaves and roots of AP have been used to cure and exhibit pharmacological activities in experimental studies and traditional medicinal systems, for examples, dysentery, enteritis, helminthiasis, herpes, peptic ulcer, skin infections (topical use), snake-bites (topical use) in India [12]. In Bangladesh, AP has been used to treat acute diarrhea, constipation, cough, diabetes, fever, headache, liver disorders, and malaria [10]. AP is commonly used for the treatment of fever, noninfectious diarrhea, and prevention of the common cold in Thailand [13]. Moreover, it was used to treat respiratory infections, pharyngitis, pneumonia, tonsillitis, and vaginitis in China [11]. Furthermore, numerous clinical studies of AP have been tested in different countries. In Thailand, AP was administered at a dose of 6 g/day for 7 days to 152 Thai adults who suffering from pharyngotonsillitis. The AP showed efficiency for symptomatic relief of acute respiratory tract infections in adults and children [14]. The previous study reported that the ethanolic extract of AP was more effective than furazolidone or chloramphenicol in treatment of acute bacillary dysentery and acute gastroenteritis [15]. The cured rate of AP was reported to be 88.3% and 91.3% for acute bacillary dysentery and acute gastroenteritis cases, respectively.

Andrographolide (AG, C_20_H_30_O_5_), is the major active principle isolated from AP. AG has been known to have therapeutically and biologically active properties. Furthermore, AG also has a similar size to estrogen and may possess estrogen-like activities [16]. Many studies have focused on AG’s activity, for examples, anticancer [17,18], hepatoprotective [19], and anti-inflammatory properties, in both in vitro and in vivo [20]. Moreover, it has been reported that AG has both stimulating effects on osteoblastogenesis [21] and inhibiting effects on osteoclastogenesis by inhibiting nuclear factor kappa-B (NF-κB) and extracellular-signal-regulated kinase (ERK) signaling pathways in vitro and in vivo [22,23]. AG has been performed in cartilage explants to the in vitro potent chondroprotective activities and therapeutic use in equine degenerative joint diseases [24] and showed a protective effect on oxidative stress in chondrocytes injured by H_2_O_2_ [25]. Thus, AG is promising to treat the progressive loss of bone and cartilage which is a main sign in early stage of OA diseases. AG has been reported to have a potential to treat periodontal disease [26] and cardiovascular disease in obese patients [27] as well. In addition, the clinical studies have reported AG administration significantly improved the CD4+ lymphocyte count in HIV- positive patients [28] and inhibited the gp120-mediated cell fusion of HL2/3 cells with TZM-bl cells [29]. Moreover, publications related with the effects of natural molecules on osteogenesis of stem cells has been currently increased [30,31,32]. Stimulating bone and cartilage formation with biologically active pharmaceuticals derived from natural sources is a direction worth investigating for the treatment of homeostasis of bone diseases and/or bone remodeling in regenerative medicine field. Due to the lack of cytotoxicity and evidence of the effects of AG on multilineage differentiation in human mesenchymal stem cells, this study is aimed to investigate the effects of AG on the in vitro cytotoxicity, and human mesenchymal stem cell differentiation in order to identify potential mechanisms of action.

## 2. Results

### 2.1. Isolation and Culture Expansion of SPFP-MSCs

**Figure 1:**
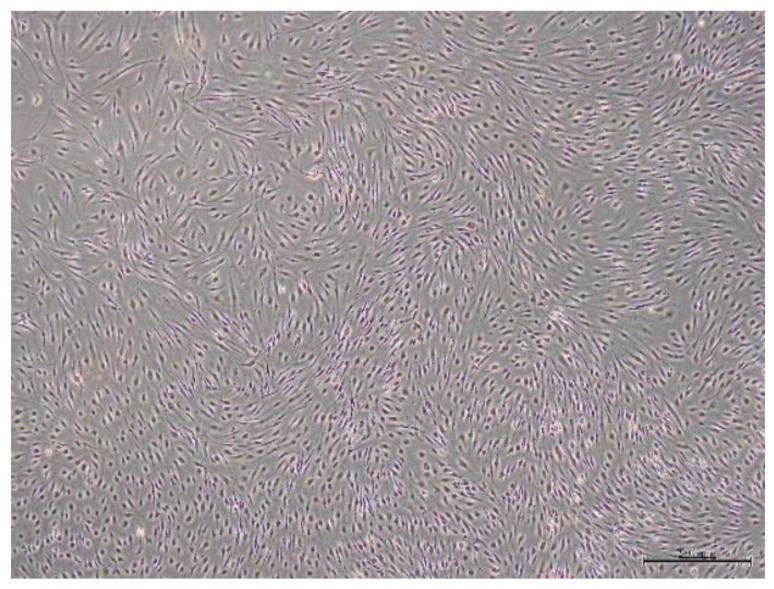
Cells morphology from SPFP tissue at 21st day of 1st passage under light microscope (4×).

Cells from SPFP samples were isolated by enzymatic digestion. After cultures had been incubated for 3 days, the isolated cells markedly spread out and adhered to the plastic culture flask. By 4–5 days, cells exhibited homogenous spindle-shaped, fibroblast-like morphology. As time went on, the cells evinced a high proliferation rapidly and took on a clustered appearance. Cultures reached 80–90% confluence in 7–10 days. Following the first sub-culturing, approximately 4–5 days were needed for each passage. Cell morphology remained homogeneous until being terminated (Figure 1).

### 2.2. Cell Surface Marker Profile

**Figure 2:**
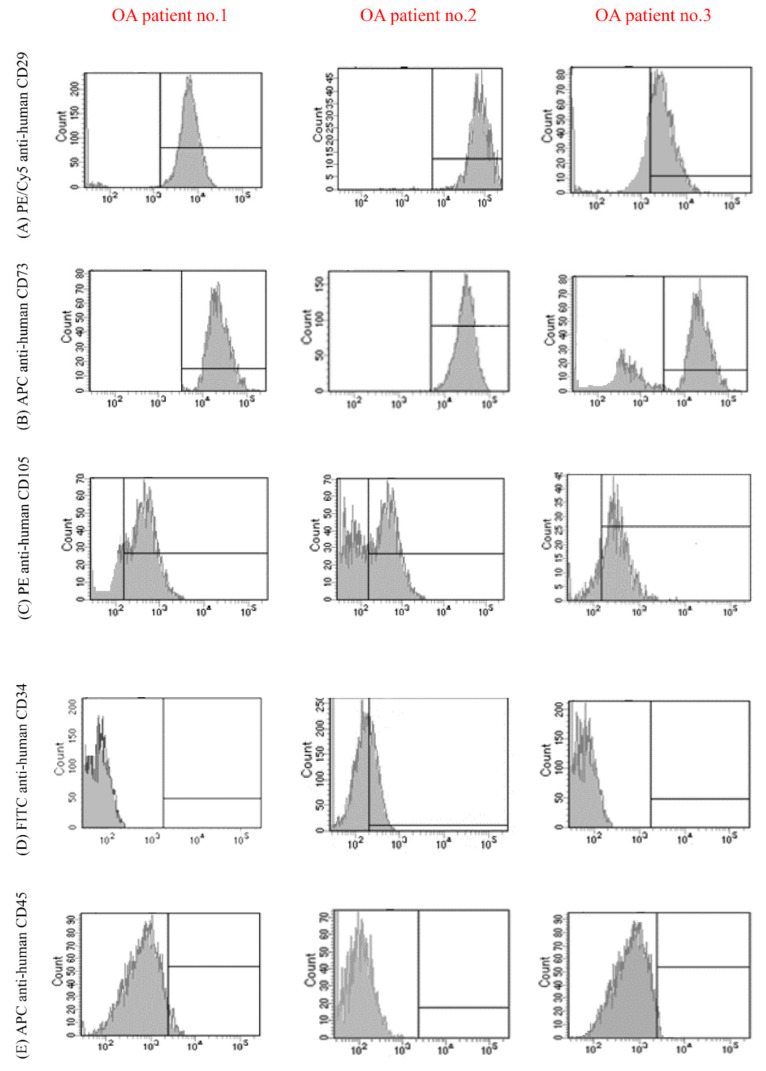
Flow cytometry analysis of the expression of cell surface markers protein in SPFP-MSCs from three OA patients. Cell cultures were positive for the MSC specific markers (**A**) CD29 = 92.30 ± 3.20%, (**B**) CD73 = 90.91 ± 2.73%, and (**C**) CD105 = 83.52 ± 3.23%. and negative for the hematopoietic stem cell markers (**D**) CD 34 = 1.04 ± 2.88% and (**E**) CD45 = 0.92 ± 1.08%.

Flow cytometry analysis demonstrated that cell populations derived from SPFP were entirely or partly positive for the MSCs, mean surface immunophenotype expression value were showed as CD29 = 92.30 ± 3.20%, CD73 = 90.91± 2.73%, and CD105 = 83.52 ± 3.23% (Figure 2A–C). Moreover, cell populations showed lacked surface expression of hematopoietic stem cell markers CD 34 = 1.04 ± 2.88% and CD45 = 0.92 ± 1.08% (Figure 2D,E).

### 2.3. Effect of AG on the Cell Viability of Differentiating SPFP-MSCs

**Figure 3:**
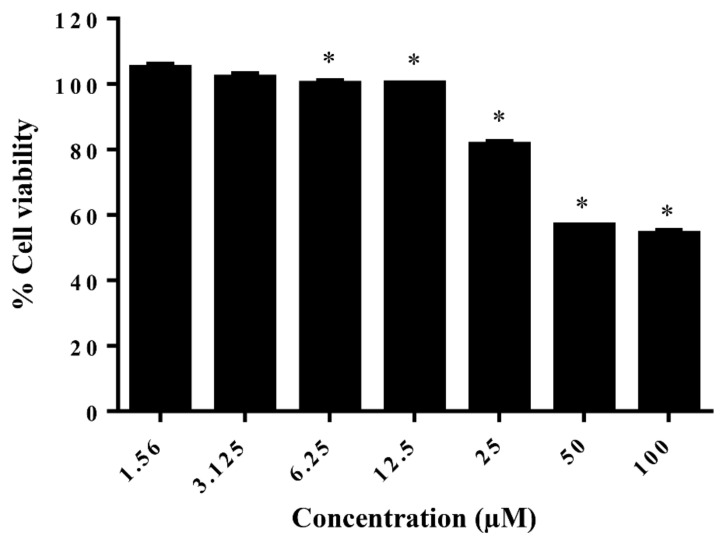
The effect of AG on cell viability of cells from SPFP-MSCs. The bar means SD. Data is shown as mean ± SD (*n* = 10). * Significant difference, *p*-value ≤ 0.05.

To investigate the effect of AG on cell viability, the MTT assay was performed by measuring mitochondrial reducing activity. The result showed that the OD values of SPFP-MSCs were stable following treatment with AG which did not influence cell viability when it was used at concentrations between 1.56 and 12.5 µM, indicating that cytotoxicity was undetectable when the concentration of AG was less than 12.5 µM. However, AG demonstrated the ability to decrease cell viability significantly at dosages above 12.5 µM, suggesting that high concentrations of AG could inhibit cell proliferation of SPFP-MSCs (Figure 3).

### 2.4. In Vitro Tri-Lineage Differentiation and Histological Analysis

First-passage of cells cultures were subjected to in vitro differentiation assays in order to evaluate their mesenchymal multipotent potential. The shape of the cells changed to a spindle-shaped morphology. Cells demonstrated a potential to differentiate to varying degrees as shown by positive osteogenic, adipogenic, and chondrogenic staining.

**Figure 4:**
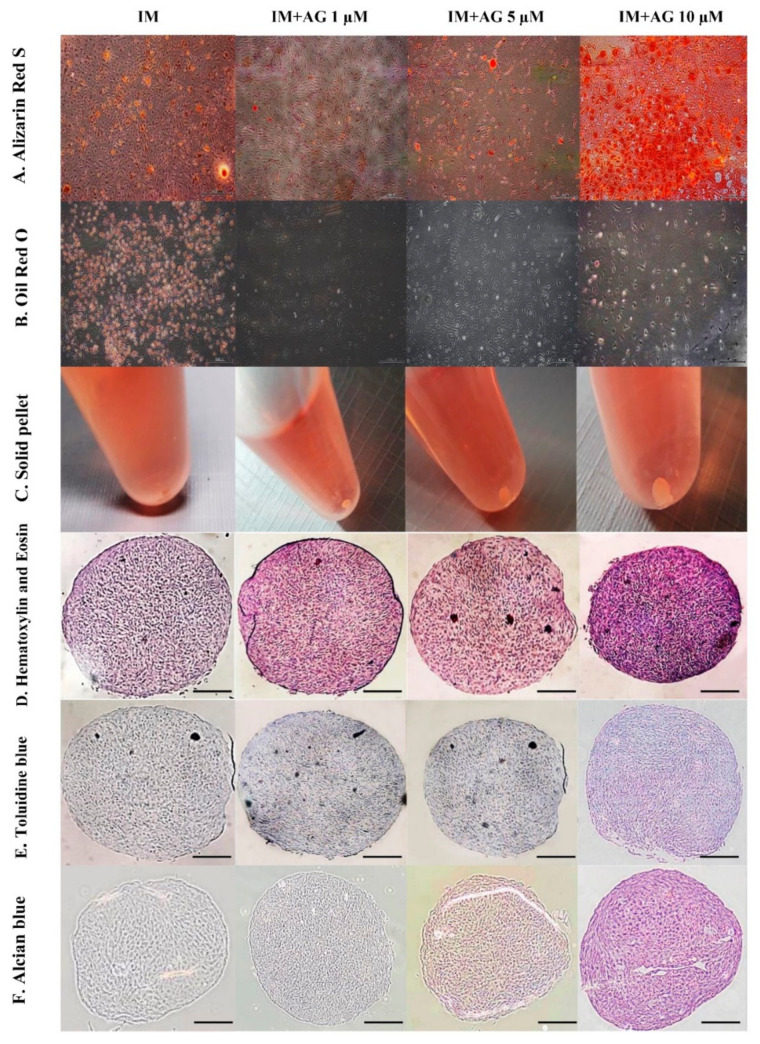
The effects of different doses of AG (1, 5 and 10 μM) with specific induction medium (IM) of each differentiation type on tri-lineage differentiation potentials of cells from SPFP-MSCs on day 21 compared with specific induction medium without AG as control. The scale bar (▬▬) in (**A**,**B**,**D**–**F**) represents 500 µm and 200 µm, respectively. (**A**) Osteogenic differentiation; Alizarin red S staining reflected that the calcium droplets. (**B**) Adipogenic differentiation; Oil Red O staining reflected that lipid droplets. (**C**) Chondrogenic differentiation; After a cell pellet was treated with chondrogenic medium and chondrogenic medium with AG. (**D**) Pellet cultures of SPFP-MSCs were sectioned and stained with Hematoxylin & Eosin reflected that morphology. (**E**) Pellet cultures of SPFP-MSCs were sectioned and stained with toluidine blue reflected that glycosaminoglycans. (**F**) Pellet cultures of SPFP-MSCs were sectioned and stained with alcian blue reflected that proteoglycans.

**Figure 5:**
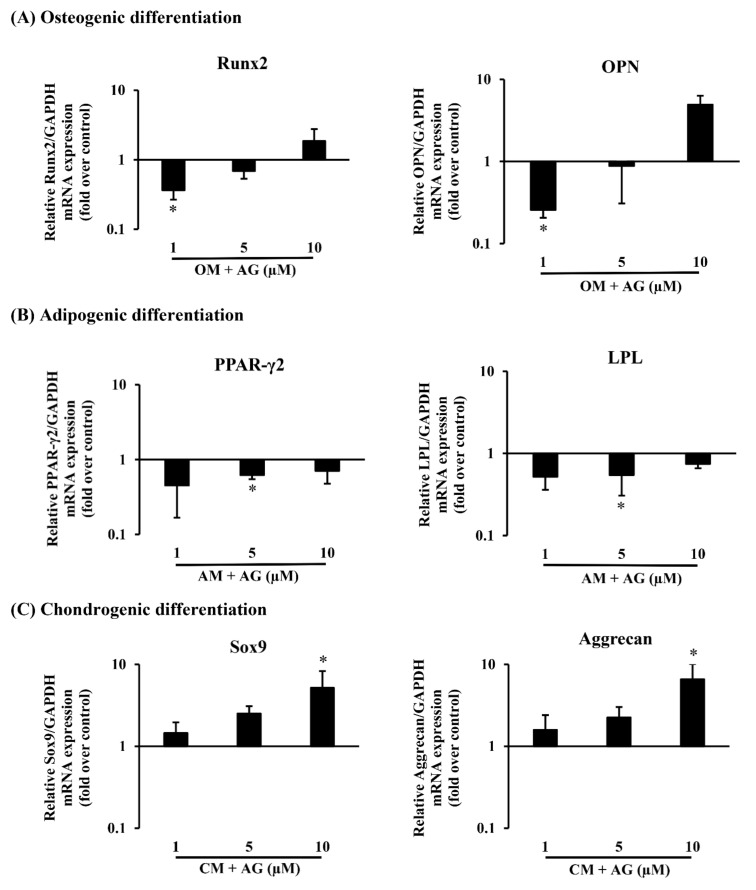
The effects of different doses of AG (1,5 and 10 µM) on tri-lineage differentiation potentials of cells from SPFP tissues on day 21. (**A**) Osteogenic differentiation: (Runx2, OPN), (**B**) Adipogenic differentiation: (PPAR-γ2, LPL), (**C**) Chondrogenic differentiation: (Sox9, Aggrecan). Gene expression of tri-lineage potentials was assessed using real-time PCR. The bar means SD. Data is shown as mean ± SD (*n* = 10). Statistical significance was calculated using a one-way ANOVA test and significance is represented on graphs as * *p*-value < 0.05.

In osteogenesis differentiation, cells seeded at 5 × 104 cells per well were seeded in 6 well plates in triplicate in culture medium. After 24 h, confluent cell populations (90% confluency) from SPFP-MSCs were promoted by treating cells cultures with osteogenic induction medium (OM) containing AG at different concentrations (1, 5, and 10 μM) while cells maintained in OM were used as controls. After 3 weeks, cells were stained with alizarin red S to confirm the accumulation of calcium (Figure 4A). Furthermore, their osteogenic potency was confirmed by examining mRNA expression of several osteogenic-specific marker genes including Runt-related transcription factor 2 (Runx2) and Osteopontin (OPN) (Figure 5A). OM containing AG 10 µM profoundly upregulated the mRNA expression of osteogenic markers compared to control groups of cells.

Using these isolated cells from SPFP, we further determined the adipogenic potential of cells with cultures using adipogenic induction medium (AM), these cells became more elongated and flatter. 5 × 10^4^ cells per well were seeded in 6 well plates in triplicate in expansion medium. After 24 h, confluent cell populations (90% confluency) from SPFP were promoted by treating expansion medium cultures with AM containing AG at different concentrations (1, 5, and 10 μM). SPFP-MSCs were observed, especially at the sites where lipid droplets became visible and compared with SPFP-MSCs maintained in AM for 3 weeks as control groups. After 3 weeks, lipid droplets were confirmed by oil red O staining to visualize accumulated cytoplasmic lipid rich vacuoles (Figure 4B). According to real-time PCR assay, expression levels of adipogenic-specific genes for peroxisome proliferator-activated receptor gamma 2 (PPAR-γ2) and lipoprotein lipase (LPL) (Figure 5B), the mRNA expression in induced groups with AM containing AG at all concentrations (1, 5, and 10 μM), especially 5 µM (*p*-value < 0.05), was lower than that in control groups.

Further analysis made was to evaluate chondrogenic differentiation capacity (pellets) of isolated MSCs from SPFP. Pellets were either introduced to CM containing AG at different concentrations (1, 5, and 10 μM) or CM with 1% FBS. The cells had grown and formed dense spheroidal pellets (Figure 4C). After 21 days, the spheroidal pellets were easily processed for staining with H&E (Figure 4D), Toluidine Blue (metachromatic) (Figure 4E) and Alcian Blue (Figure 4F). The results showed morphology as demonstrated by H&E, the presence of glycosaminoglycans as demonstrated by toluidine blue and the presence of enrichment of sulphated proteoglycans as illustrated by alcian blue staining. The mRNA expression levels of chondrogenic-specific genes including SRY-related transcription factor 9 (Sox9) and Aggrecan (Figure 5C) in the induction pellets containing AG at all concentrations (1, 5, and 10 μM), especially 10 µM (*p*-value < 0.05), was higher than those in the CM as control group.

## 3. Discussion

The number of elderly patients in need of treatment related to bone formation has increased, and they may have a decreased capability of bone remodeling. Therefore, stimulating bone and cartilage formation from natural sources that selectively enhance osteogenesis and chondrogenesis without stimulating adipogenesis are desirable.

AP has long been used in the traditionally known medical systems in Asia. In Thailand, this medicinal plant was selected by the Ministry of Public Health as one of the medicinal plants to be recorded in “The National List of Essential Drugs A.D. 1999” (List of Herbal Medicinal Products [33]). Several studies have shown that its main bioactive component, AG, has a broad range of beneficial pharmacological effects, such as antiviral, anti-malarial, immunostimulatory, and anti-inflammatory. Moreover, it contains bioactive molecules like estrogen. In the present study, we have significant findings. We have demonstrated that AG did not influence cell viability when it was used at concentrations ranging from 1.56 to 12.5 μM. This indicates that AG did not have an effect on human mesenchymal stem cells. In addition, we also originally demonstrated for the first time that AG promoted osteogenic and chondrogenic differentiation of SPFP-MSCs and suppressed their adipogenic differentiation.

There are two major modes of osteogenesis, and both involve the transformation of a preexisting mesenchymal tissue into chondroblast, chondrocytes, and osteoblasts. The mesenchymal stem cells differentiate into chondroblasts and chondrocytes and this cartilage is later replaced by bone. The important markers for osteogenic differentiation, Runx2 and OPN, where Runx2 is a master regulator in the late-stage marker of osteogenesis and OPN is the important intermediate stage marker during the differentiation into mature osteoblasts for osteogenic differentiation followed by matrix maturation and matrix mineralization [34,35,36]. The previous study also showed that AG exhibits the inhibitory effects on osteoclastogenesis and osteoclast function in vitro and in vivo through the suppression of nuclear factor-kappaB (NF-κB) and extracellular-signal-regulated kinase (ERK) signaling pathways [22]. After 21 days of culture with SPFP-MSCs, AG stimulated the expression of Runx2, OPN and increased the calcium deposition activity in a dose-dependent manner, suggesting that AG could enhance the osteogenic ability of SPFP-MSCs.

In order to investigate whether AG could inhibit adipogenic differentiation of SPFP-MSCs, the adipocyte-specific markers including PPAR-γ2 and LPL and also qualitative of adipocytes number (Oil Red O staining) were examined. The PPAR-γ2 which has been shown to play an important role in mature adipocyte differentiation as a terminal differentiation marker, lipid storage, insulin sensitization and can be activated by fatty acids [37]. LPL is thought to be an early marker of adipogenesis and highly expressed during adipogenic differentiation [38]. As shown in this study, after 21 days of culture treatment with AG. AG suppressed both PPAR-γ2 and LPL mRNA expression by inhibiting adipocyte cells formation. These results suggested that AG could suppress the adipogenic differentiation both qualitatively and quantitatively.

In this study, we used an in vitro cell pellet culture of chondrogenesis for 21 days derived from SPFP-MSCs and found the different concentrations of AG were able to significantly enhance differentiation in a dose-dependent manner by upregulating a number of genes associated with chondrogenesis such as Sox9 and Aggrecan under chondrogenic plus AG extract conditions [39]. Moreover, different concentrations of AG revealed a significant higher cell growth rate than the control. In addition, qualitative study exhibited increased synthesis of matrix proteoglycans and visible formation of glycosaminoglycan when stained with toluidine blue and alcian blue [40]. The resulting pellet was fixed with H&E stain for the identification of chondrocyte morphology and revealed a significant dark stain in doses dependent of AG. The intensities of the stain were dependent on the dose of AG.

## 4. Materials and Methods

Andrographolide (C_20_H_30_O_5_, >98% purity, Lot No. C7OZC-PF) was purchased from Tokyo Chemical Industry Co., Ltd., (Tokyo, Japan). Cultures of human tissue samples were collected from the suprapatellar fat pad (SPFP) tissue as surgical waste from ten osteoarthritis patients aged over 60 years who underwent total knee arthroplasty (TKA). Informed consent was obtained before surgery. The study protocol conformed to the guidelines of the Declaration of Helsinki and was approved by the Institution Review Board (MU-DT/PY-IRB 2018/037.1309) at Faculty of Dentistry/Faculty of Pharmacy, Mahidol University, Bangkok, Thailand prior to perform the study.

### 4.1. MSCs Isolation, Expansion and Culture

SPFP tissues were harvested during TKA with tissues and then used for cell isolation, and for collecting mesenchymal stem cells (MSCs) according to a previously published protocol [41]. In brief, SPFP tissues from resections were rinsed and washed with phosphate buffered saline (PBS, Gibco™, Life Technologies, Grand Island, NY, USA), and then chopped in to small pieces. After that, 0.1% collagenase type I solution (Gibco™, Life Technologies, Grand Island, NY, USA) was added and incubated under warm-water bath (37 °C) for 60 min then suspended with Dulbecco’s Modified Eagle Medium-High glucose (DMEM-HG) (Gibco™, Life Technologies, Grand Island, NY, USA) containing 10% fetal bovine serum (FBS) (Gibco™, Life Technologies, Grand Island, NY, USA) and centrifuged at 400× *g* for 10 min and the surfactant layer was removed. The resulting pellet was mixed with 15 mL of complete medium (DMEM-HG + 10% FBS + 1% L-glutamic acid (Gibco™, Life Technologies) + 1% Pen-strep (Gibco™, Life Technologies, Grand Island, NY, USA), then passed through a sterile filter/cell strainer (Corning Inc., Corning, NY, USA) and finally seeded in T-175 tissue culture flasks (Wuxi NEST Biotechnology, Nanjing, China) which were placed in a 37 °C incubator with 5% CO_2_. Culture medium was replaced every 3 days. If cells expanded to more than 80% of the culture flask, then SPFP cells were detached with 0.05% Trypsin/0.1% EDTA (Gibco™, Life Technologies, Grand Island, NY, USA) and recultured as the first passage with complete medium through 2nd passage. Cell count and time between each passage were recorded. Suprapatellar fat pad derived mesenchymal stem cells (SPFP-MSCs) in the first passage were trypsinized and divided for the following assays following International Society for Cellular Therapy (ISCT).

### 4.2. Flow Cytometric Analysis

The SPFP-MSCs were assayed for cell immunophenotype surface protein expression and SPFP-MSCs must express CD29, CD73 and CD105, and lack expression of hematopoietic linage markers CD45, CD34 according to a previously published articles [42,43,44] by flow cytometry (FACSverse™, Beckman Coulter Inc., Brea, CA, USA). The 10 μL of cell suspension with density at 5 × 10^6^ cells was diluted into 200 μL of PBS and centrifuged at 900× *g* for 3 min. Then, the cell supernatant was discarded, added with 1 μL of antibody, and mixed by vortex. Cells were incubated for 30 min at 4 °C in the dark at room temperature. Then, cells were fixed by addition of 50 μL 4% paraformaldehyde in PBS for 15 min in the dark at room temperature and centrifuged at 900× *g* for 3 min. The supernatant was discarded and resuspended in 0.3 mL of PBS for FACS analysis. As a negative control, cell suspension without antibody was employed following the same procedure.

### 4.3. Cell Viability Assay

SPFP-MSCs from passage 1 were seeded into 96-well plates at a density of 1 × 104 cells/well in triplicate for 24 h and then the cells were incubated with control blank medium (fresh DMEM) and blank medium with increasing concentrations of AG (1.56, 3.125, 6.25, 12.5, 25, 50 and 100 μM) for 24 h. Next, the cells in each well were washed with PBS and replaced by 100 μL of 0.5 mg/mL MTT solution. The cells were then incubated for another 4 h at 37 °C under 5 % CO_2_, after which the MTT solution in each well was carefully discarded and replaced by 50 μL of dimethyl sulfoxide (DMSO) to dissolve formazan crystals. The optical density (OD) was then measured with multimode microplate reader (Infinite M200 PRO, Tecan Group Ltd., Männedorf, Switzerland) at a wavelength of 590 nm. The cell viability was calculated relative to the control using the following formula:

(1) $$\mathrm{Cell}\;\mathrm{viability}=\;\frac{\mathrm{sample}\;\mathrm{group}\;\mathrm{OD}\;-\;\mathrm{zeroing}\;\mathrm{OD}\;}{\mathrm{control}\;\mathrm{group}\;\mathrm{OD}\;-\;\mathrm{zeroing}\;\mathrm{OD}}$$

### 4.4. In Vitro Tri-Lineage Differentiation and Histological Analysis

Passage 1 SPFP-MSCs samples from ten knee osteoarthritis patients were subjected to directed differentiation to induce osteogenesis, adipogenesis, and chondrogenesis (Table 1). All directed-differentiation media were freshly prepared in house.

Osteogenesis: SPFP-MSCs were seeded at a cell density of 5000 cells/cm^2^ in osteogenic medium, prepared freshly in house and containing 10 nM dexamethasone, 10 mM β-glycerophosphate and 50 μg/mL ascorbate-2 phosphate (Sigma-Aldrich, St. Louis, MO, USA) and with 10% FBS. SPFP-MSC cultures were maintained into AG containing media at various concentrations (1, 5, and 10 μM) for 21 days. Medium was changed every 3 days. The extracellular calcium deposits were determined from 100% ethanol-fixed cell cultures by incubating with 0.2% *w/v* Alizarin Red S solution for 40 min at room temperature. Excess dye was removed by several washing steps using water and observed under an inverted light microscope.

Adipogenesis: SPFP-MSCs were seeded at a cell density of 5000 cells/cm^2^ and when the SPFP-MSCs were >90% confluent, the growth medium was substituted with in-house, freshly prepared differentiation medium containing 10 µg/mL insulin, 100 nM dexamethasone, 0.45 mM 3-isobutylmethylxanthine (IBMX), and 50 µg/mL indomethacin (Sigma-Aldrich, St. Louis, MO, USA). The cells were then incubated in AG-containing media at varying concentrations (1, 5, and 10 μM) for 21 days. The adipogenic differentiation was evaluated from formalin-fixed cell cultures by incubating with 0.3% (*w*/*v*) oil red O (Sigma-Aldrich, St. Louis, MO, USA) solution for 15 min at room temperature. Then, the excess staining was washed and removed with water. The stained lipid vacuoles were observed under inverted light microscope.

Chondrogenesis: The SPFP-MSCs were cultured to form a cell pellet in a 15 mL polypropylene tube (Wuxi NEST Biotechnology). Approximately 2 × 105 cells were cultured in chondrogenic medium (CM) with in-house, freshly prepared medium with the presence of 10 nM dexamethasone (Sigma-Aldrich), 10 ng/mL transforming growth factor-β3 (TGF-β3) (ProSpec, Rehovot, Israel) and 6.25 μg/mL insulin-transferrin-selenium (ITS supplement) (MP Biomedicals LLC, Irvine, CA, USA) were expanded. The cells were then incubated with andrographolide-containing media at different concentrations (1, 5, and 10 μM) for 21 days with medium changes every 3 days. Alcian blue, Toluidine Blue, and Haematoxylin & Eosin (H&E) were used to detect the presence of enrichment of glycosaminoglycans in cartilage, polysaccharides and morphology, respectively. Before staining, the cell pellet cultures were fixed in formalin then embedded in paraffin and sectioned into 4–5 μm and stained with H&E, Alcian Blue and Toluidine Blue. The sections were observed under an inverted light microscope.

### 4.5. RNA Isolation and Real-Time PCR Analysis

The differences between the messenger RNA (mRNA) levels of osteogenic, adipogenic and chondrogenic related genes in differentiation of stem cell treated without and with andrographolide at different concentrations (1, 5, and 10 μM) were analyzed using real-time PCR. Total RNA was extracted on days 0 and 21st of each induction period using a FavorPrep Blood/Cultured Cell Total RNA Mini Kit (FAVORGEN, Ping-Tung, Taiwan) according to the manufacturer’s instructions. Real-time PCR was conducted using SensiFAST^TM^ SYBR^®^ Lo-ROX one step kits. Quantitative mRNA analysis was performed by the Mx 3005P Real Time PCR system (Stratagene, Agilent Technologies Germany GmbH & Co. KG, Baden-Württemberg, Germany). Specific primer pairs for each gene as shown in Table 2. The expression level of each gene level was calculated by the comparative cycle threshold (CT) method and normalized with that of the housekeeping gene, glyceraldehyde-3-phosphate dehydrogenase (GAPDH). A comparison of mRNA expression in each sample was calculated based on the differences in ΔCT of individual samples (ΔΔCT). Graphs show the relative expression levels compared with the control on day 0.

### 4.6. Statistical Analysis

Statistical analyses were performed using SPSS version 20.0 (IBM Corp, Armonk, NY, USA). All Data were expressed as the mean ± standard deviation (SD). *p*-value of < 0.05 was considered statistically significant in this study. Analysis of variance was calculated for comparison in cell viability, in vitro tri-lineage differentiation, and gene expression of SPFP-MSCs in each source.

## 5. Conclusions

In conclusion, this study is the first to demonstrate that AG in the concentration where cell viability was more than 90% could promote coupled cell proliferation. Moreover, AG could promote osteogenic and chondrogenic differentiation in a dose-dependent manner whereas inhibit their adipogenesis of SPFP-MSCs. Furthermore, AG is associated with regulation of osteocyte-specific markers including Runx2, OPN and chondrocyte-specific markers including Sox9 and Aggrecan activity, and its anti- adipogenic effect is associated with blocking PPAR-γ2 and LPL signaling. Our data also strongly suggested that AG could be developed and expected for the possibility to translate the results to human clinical practice as a treatment for regenerative medicine for cartilage and bone regeneration. Moreover, our laboratory has foreseen the possible new class of therapeutic agents similar to AG. Thus, we are continuously investigating similar effect of molecules that have structural like estrogen from Thai medicinal herb on mesenchymal stem cells. The mechanistic insights into biological activities of these molecules are explored.

## Figures and Tables

**Table 1:** Baseline characteristics of patients in the current study.

Patient No.	Age	Sex	Diagnosis
1	81	Female	Osteoarthritis
2	75	Male	Osteoarthritis
3	61	Female	Osteoarthritis
4	78	Female	Osteoarthritis
5	76	Female	Osteoarthritis
6	86	Female	Osteoarthritis
7	77	Female	Osteoarthritis
8	83	Female	Osteoarthritis
9	75	Female	Osteoarthritis
10	81	Female	Osteoarthritis

**Table 2:** Primer oligonucleotide sequences used for real-time PCR.

Gene	Forward Primer 5-3	Reverse Primer 5-3
Runx2	5′TATGGCACTTCTTCAGGATCC′3	5′GCGTCAACACCATCATTCTGG′3
OPN	5′TGAAACGAGTCAGCTGGATG′3	5′TGAAATTCATGGCTGTGGAA′3
PPAR-γ2	5′GCTGTTATGGGTGAAACTCTG′3	5′ATAAGGTGGAGATGCAGGCTC′3
LPL	5′GAGATTTCTCTGTATGGCACC′3	5′CTGCAAATGAGACACTTTCTC′3
Sox9	5′ATCTGAAGAAGGAGAGCGAG′3	5′TCAGAAGTCTCCAGAGCTTG′3
Aggrecan	5′TGAGGAGGGCTGGAACAAGTACC′3	5′GGAGGTGGTAATTGCAGGGAACA′3
GAPDH	5′GTGAAGGTCGGAGTCAACGG′3	5′TCAATGAAGGGGTCATTGATGG′3

## Data Availability

Not applicable.

## Sample Availability

Samples of the compounds are not available from the authors.
